# Oncocytic Adrenocortical Neoplasm with Concomitant Papillary Thyroid Cancer

**DOI:** 10.3389/fendo.2017.00384

**Published:** 2018-01-22

**Authors:** Michele Podetta, Marc Pusztaszeri, Christian Toso, Michel Procopiou, Frédéric Triponez, Samira Mercedes Sadowski

**Affiliations:** ^1^Department of Visceral Surgery, University Hospitals of Geneva, Geneva, Switzerland; ^2^Department of Clinical Pathology, University Hospitals of Geneva, Geneva, Switzerland; ^3^Private Practice, Endocrinologist, Neuchâtel, Switzerland; ^4^Department of Thoracic and Endocrine Surgery, University Hospitals of Geneva, Geneva, Switzerland

**Keywords:** adrenal oncocytoma, papillary thyroid cancer, hereditary cancer syndrome, functional adrenal neoplasm, concomitant neoplasia

## Abstract

Adrenal oncocytoma (AO) is an extremely rare adrenocortical neoplasm and little is known about its malignant potential, secretory properties, and hereditary origin. We present the case of a benign AO with concomitant incidentally found papillary thyroid cancer (PTC) and review similar cases in the literature. Immunohistochemistry and next-generation sequencing (NGS) were performed. A 66-year-old women was incidentally found to have a large, androgen-secreting right adrenal mass. ^18^F-Fluorodeoxyglucose positron emission tomography showed intense uptake (SUV_max_ 88.7) of this mass and found a hypermetabolic right thyroid mass. Open adrenalectomy was performed for this highly suspicious adrenal mass. Histopathology revealed benign AO that was BRAF^V600E^ negative, with low Ki-67, and no somatic mutation found on NGS. Thyroidectomy revealed invasive, BRAF^V600E^-positive PTC. At 6 months follow-up, androgen levels returned to normal, and no recurrence was seen on imaging. To our knowledge, this is the first report of an androgen-secreting AO with concomitant PTC. Possibly the simultaneous discovery of two independent neoplasms was observed. In conclusion, this case highlights that care should be given to exclude concomitant neoplasms. Long-term and regular imaging with biochemical follow-up is warranted, since the outcome and clinical behavior of AO remains uncertain.

## Background

Oncocytic neoplasms occur in various organs such as the kidneys, thyroid, parathyroid, or salivary glands. An adrenal location, called adrenal oncocytoma (AO), is extremely rare with about 150 reported cases. They have been considered benign non-functional neoplasms; however, in about 20% of cases, they show malignant potential, and in 15% of cases, adrenocortical hormone secretion was detected (1). In most cases, these adrenal masses were detected incidentally at a large size and were thus suspicious for adrenocortical carcinoma (ACC) (1). Computed tomography (CT) scan or magnetic resonance imaging is weak in defining the nature of large adrenal masses, and the role of ^18^F-FDG positron emission tomography (PET) remains uncertain. Fine needle aspiration (FNA) biopsy does not allow for the diagnosis of AO, and since their biological behavior is uncertain, adrenalectomy remains the only reliable diagnostic and therapeutic option.

The incidence of thyroid cancer (TC) is about 3 cases per 100,000 population, and papillary thyroid cancer (PTC) represents 85% of TC, with a good prognosis (2). Mutations with activation of MAPK and PI3K-AKT signaling pathways are crucial in the pathogenesis of PTC. Common alterations are point mutations of *BRAF* (most commonly V600E) and *RAS* genes as well as RET/PTC and PAX8/PPARγ chromosomal rearrangements. The type of alteration in PTC seems to be linked to specific etiologic factors. Rearrangements have a strong association with exposure to ionizing radiation and possibly DNA fragility, whereas point mutations probably arise as a result of chemical mutagenesis (3).

No endocrine syndrome is known associating AO and PTC. Given the presentation of two rare endocrine tumors in this patient, a possible hereditary link was suggested, especially when initially suspecting an ACC. None of the known syndromes caused by hereditary predisposition specifically include PTC and ACC or adrenal adenoma. Multiple endocrine neoplasia (MEN) Type 1 (*Menin* gene) is known to present with multiples combinations, including ACC but not PTC (4) and MEN Type 2 syndromes (*RET* gene) include pheochromocytoma and medullary TC, but do not traditionally include PTC or ACC. ACC is known to appear in Li–Fraumeni syndrome with *TP53* mutation. There are four reports that attribute the association of ACC and PTC to coincidence, but do not discount a potential genetic or hereditary link (5–8).

We describe a case with two incidentally found endocrine tumors, an androgen-secreting AO mimicking ACC and a PTC.

## Case Presentation

**Figure 1:**
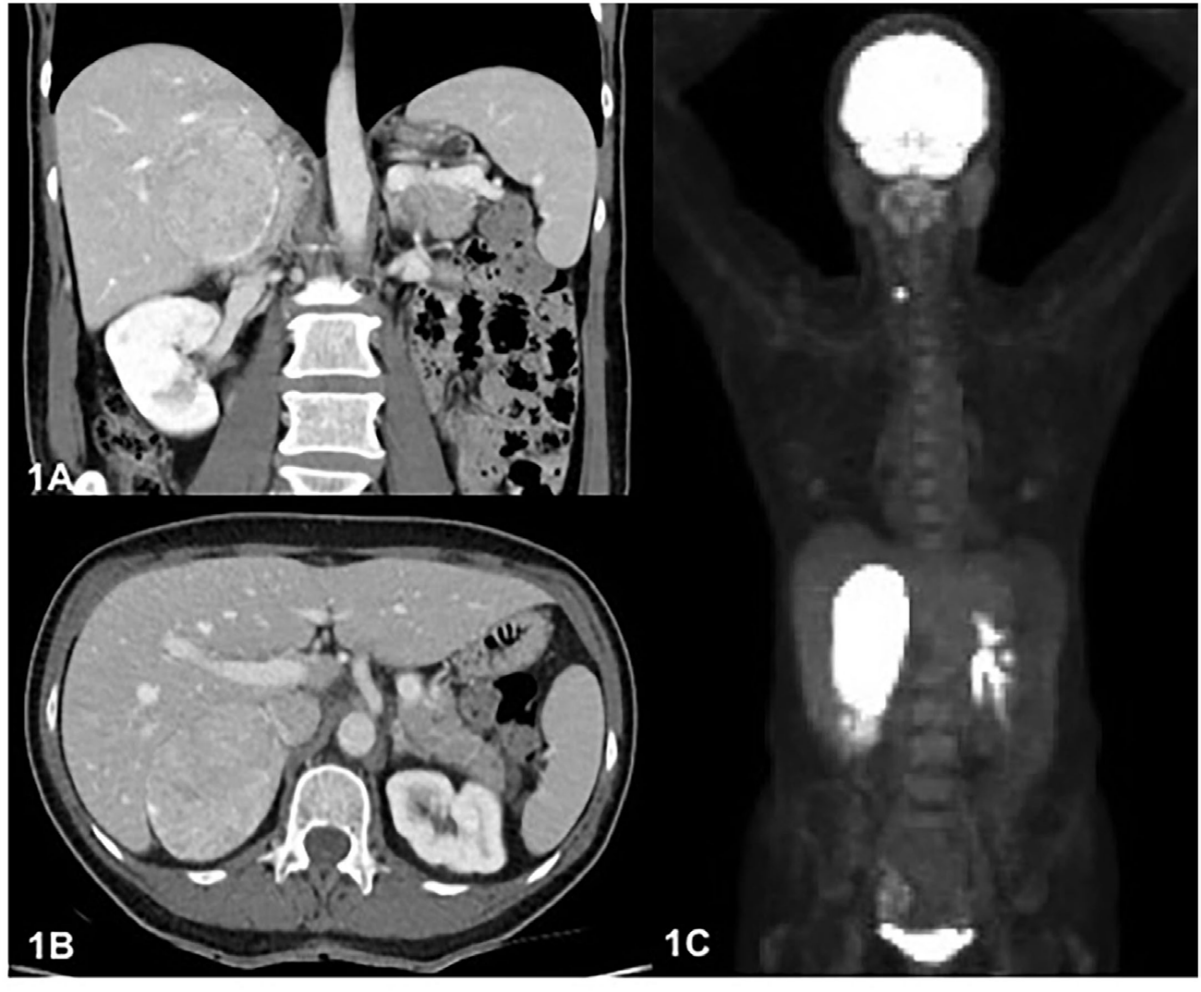
**(A)**. Axial image on portal phase computed tomography (CT) scan showing right adrenal mass heterogeneously enhanced of 7.6 cm × 5.8 cm. **(B)**. Cross-sectional image on CT scan showing right adrenal mass with imprint on the liver. **(C)**. Maximum intensity projection image on FDG positron emission tomography showing the two hypermetabolic masses, right adrenal, and right thyroid lobe.

A 66-year-old women was admitted with a complaint of transient global amnesia and abdominal pain. She mentioned a 4-year history of fatigue with occasional headaches and epigastric burn-like pain persisting for 1 month. No previous episodes of pain, weight loss, or family history for endocrine malignancy were reported. Physical examination revealed painful upper right quadrant palpation and the absence of Cushing syndrome features or hirsutism. Abdominal ultrasound (US) revealed a hypoechoic mass, and CT scan revealed a 7.6 cm × 5.6 cm × 7.4 cm right adrenal mass, hyperdense (>50 Hounsfield units) with delayed wash out after contrast injection (Figures 1A,B). The left adrenal was normal. Further endocrine work-up revealed normal cortisol after 1 mg of dexamethasone (43 nmol/l), normal 24-hour urinary excretion (156 nmol/24 h), and normal free plasma metanephrines. Dehydroepiandrosterone sulfate (DHEAS) was increased after dexamethasone suppression at 21.7 μmol/l (range, 0.9–2.0), testosterone at 2.6 nmol/l (range, 0.5–1.9), and estradiol at 237 pmol/l (<103 postmenopausal). TSH and calcium levels were normal.

**Figure 2:**
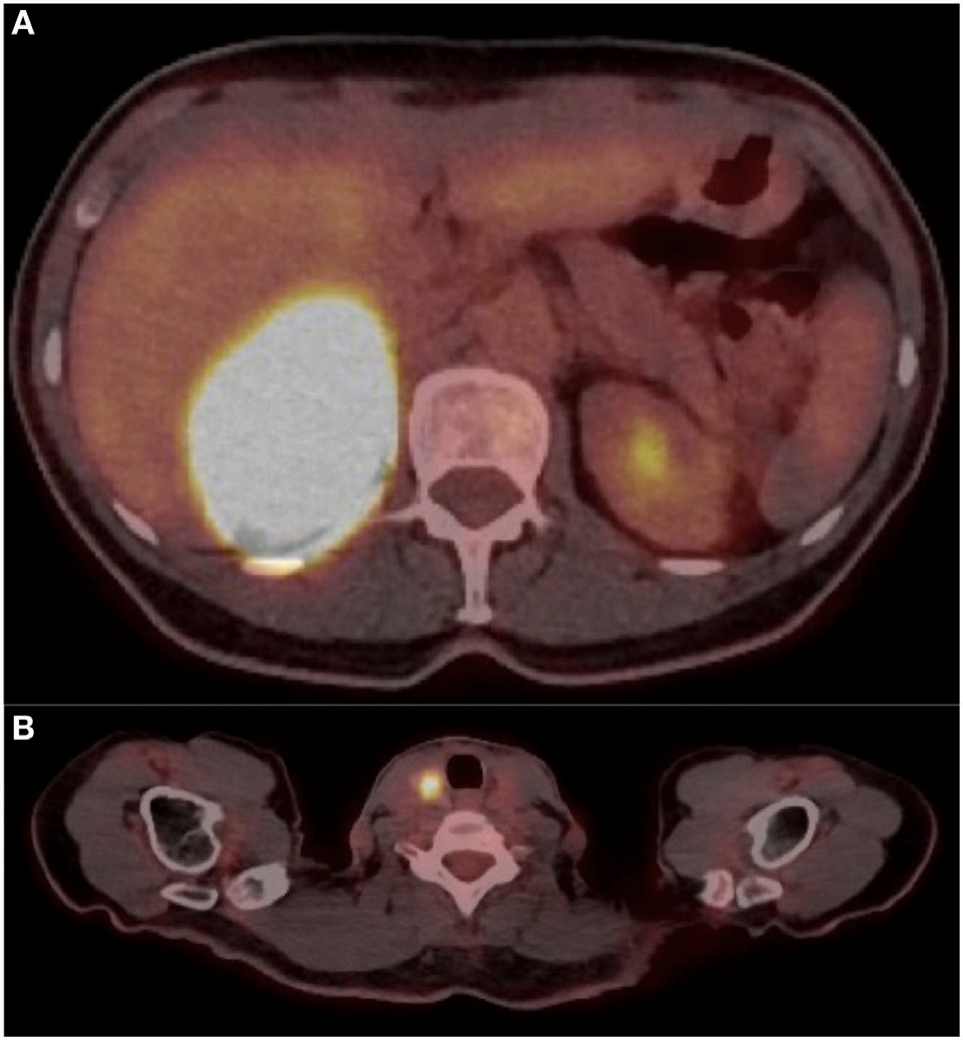
**(A)** Cross-sectional image on FDG positron emission tomography (PET) showing right adrenal mass at SUV max 88.7. **(B)** Cross-sectional image on FDG PET showing right thyroid mass at SUV max 10.6.

Because of suspected malignancy, a ^18^F-FDG PET was performed, revealing a highly metabolic 7.3 cm × 5.6 cm × 8.1 cm right adrenal mass (SUV_max_ 88.7) with a mass effect on the liver (Figures 1A–C and 2A). Hypodense areas within the mass suggested necrotic spots, however, without local infiltrative signs. In addition, a hypermetabolic right thyroid lesion of 1.2 cm was found (SUV_max_ 10.6; Figure 2B) without suspicious lymph nodes, and US-guided FNA revealed PTC.

Based on the size, hypermetabolic activity, and androgen secretion, malignancy of the adrenal mass was suspected, and an open right adrenalectomy was performed. The adrenal mass was well encapsulated, without any intraoperative signs of local invasion. Synchronous total thyroidectomy and right level VI node dissection were performed. Isthmus and left thyroid lobe were found to be absent (thyroid hemiagenesis).

**Figure 3:**
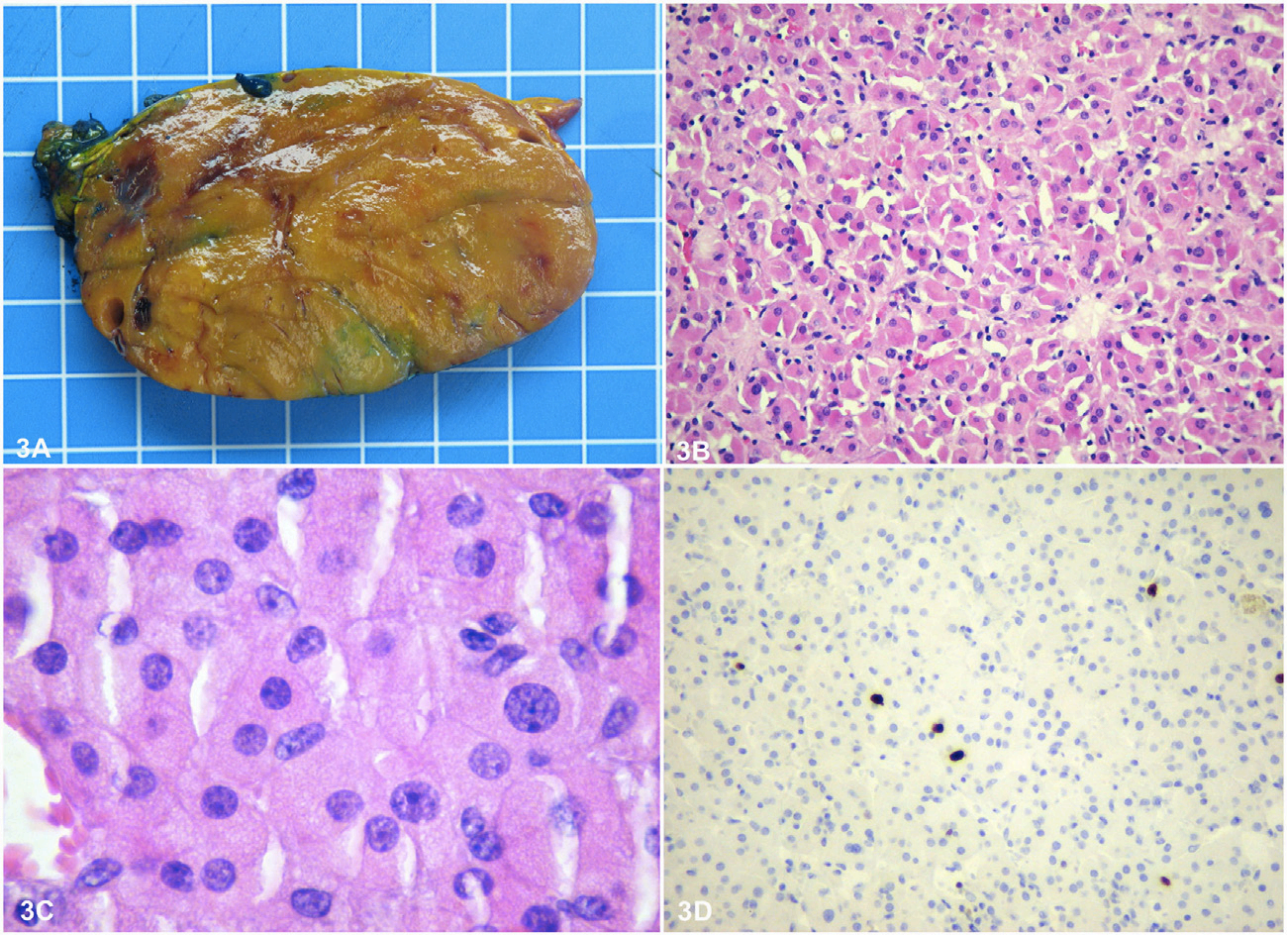
Macroscopically, the adrenal tumor was a rounded, well-circumscribed orange brown homogeneous mass, measuring 8.5 cm × 6 cm × 4.5 cm **(A)**. The size of the grid in the background is 1 cm × 1 cm. Microscopically, it was well circumscribed and partially encapsulated and consisted entirely of bland looking oncocytic cells, with granular eosinophilic cytoplasm and round nuclei with prominent nucleoli, arranged in a solid pattern **(B,C)**. Parenchymatous or venous invasion, high mitotic rate, and atypical mitoses were not observed. Therefore, according to the Lin–Weiss–Bisceglia criteria, it was diagnosed as a benign oncocytic adrenocortical adenoma. Further evaluation using immunohistochemistry revealed negative BRAF V600E immunostaining and a low Ki-67 proliferation index (<2%) **(D)**.

**Figure 4:**
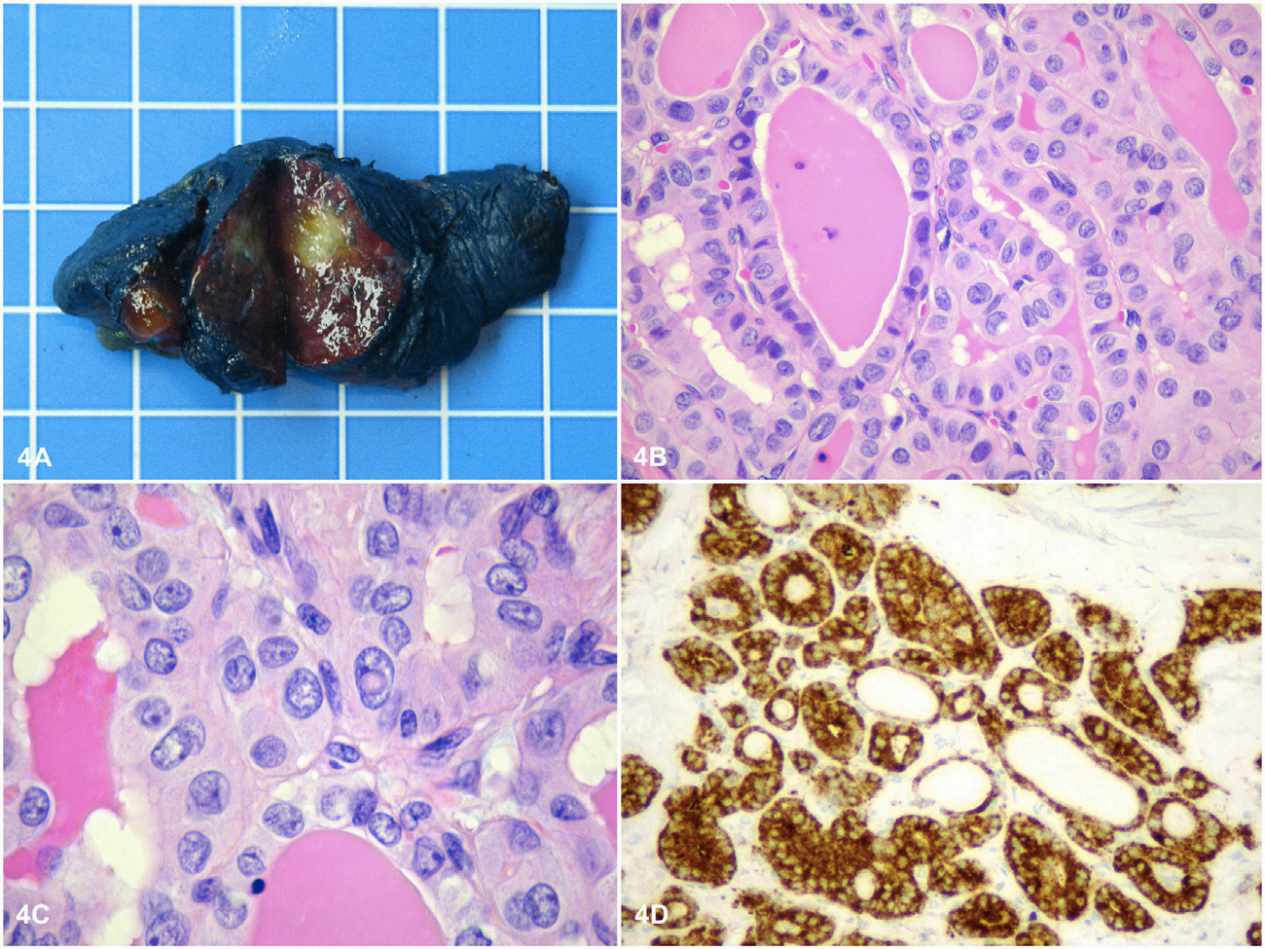
Macroscopically, the thyroid tumor was a rounded, well-circumscribed white intrathyroidal nodule measuring 1.2 cm × 1 cm × 0.8 cm **(A)**. The size of the grid in the background is 1 cm × 1 cm. Microscopically, the tumor consisted of cuboidal cells arranged mostly in follicles with a few papillae, showing classical nuclear features of papillary thyroid cancer (PTC), including chromatin pallor, numerous nuclear grooves, and several nuclear pseudoinclusions **(B,C)**. Further evaluation using immunohistochemistry revealed positive BRAF V600E immunostaining in the tumor cells **(D)**, consistent with the classical type of PTC (BRAF-like PTC).

Histopathology of the adrenal showed a rounded, well-circumscribed, and encapsulated mass, measuring 8.5 cm, without necrosis (Figure 3A). The margins were negative on examination. Microscopically, the tumor was entirely composed of oncocytic cells (Figures 3B,C). The Lin–Weiss–Bisceglia criteria were absent, and the mass was classified a benign AO. Further evaluation using immunohistochemistry revealed negative BRAF^V600E^ immunostaining and a low Ki-67 proliferation index (<2%) (Figure 3D). In addition, next-generation sequencing (NGS) was performed on the adrenal tissue, including known mutations in ACC [*p53, RB1*, and *CTNNB1* (9)]; 2,800 (hotspot) mutations in a classic panel of 50 genes associated with different cancers (Ion Ampliseq Cancer Hotspot v2) were assessed using the Ion Proton™ system (see Table S1 in Supplementary Material for list of the 50 tested genes). NGS did not reveal any mutations for this adrenal mass. Histopathology of the thyroid gland revealed an invasive PTC of 1.2 cm, BRAF^V600E^ positive, without lymphovascular invasion (Figures 4A–D), staged pT1b, N1a, R1 (3/4 positive central lymph nodes, R1: microscopically positive resection margin).

Postoperative recovery was uneventful, and patient was started on thyroid hormone replacement therapy. According to ATA guidelines (2) and local invasiveness, the patient underwent I^131^ radioactive iodine therapy. Postoperative work-up at 2 months showed normalization of DHEAS to 1.7 μmol/l and morning cortisol at 464 nmol/l, as well as testosterone level at 0.8 nmol/l. Corrected calcium was normal at 2.32 mmol/l and thyroglobulin at <0.10 μg/l. For the thyroid lesion, the patient will be followed up by measuring stimulated thyroglobulin after radioactive iodine therapy, and aiming for a suppressed TSH, as well as by performing a neck ultrasonography and thyroglobulin at 6-month intervals. For the adrenal lesion, at 6-month follow-up, the androgen levels stayed normal (1.7 μmol/l), and no recurrence was seen on CT scan imaging. Regular imaging and biochemical follow-up at 12 and 18 months for a minimum of 5 years are planned.

## Discussion

We describe a female patient with a highly suspicious adrenal mass that reveals to be a benign lesion based on the pathological criteria, without somatic mutations, and who presents with a simultaneous endocrine malignancy of the thyroid gland.

Adrenal oncocytoma is a rare neoplasm arising from the adrenal cortex. Malignant oncocytoma and ACC are large, heterogeneous, with internal necrosis and associated with slow wash out, such as found in this case’s preoperative imaging. The concern for metastatic disease led us to perform a ^18^F-FDG, which revealed a highly metabolic adrenal mass and the possibility of another synchronous primitive tumor or metastasis (Figures 1C, 2B); ultimately it led to the detection of concomitant PTC. Adrenal metastases from PTC have been described (10, 11). In reported cases, metastases were smaller than this case’s mass, and the adrenal tumors were detected by ^18^F-FDG, but were not as metabolically active (10). Little is known about the ^18^F-FDG behavior of AO. Some cases of high metabolic uptake have been reported, with uptake explained by intense activity of glucose metabolism or the high number of mitochondria known to be present in oncocytic cells (12, 13); and high uptake has been described in other oncocytic lesions such as in lung carcinoids (14).

The elements such size, hypermetabolic activity, and androgen secretion led us to suspect a malignant primary adrenal mass or a metastatic tumor, possibly invading the liver, and, therefore, open adrenalectomy was preferred for radical tumor resection (15). The safety of laparoscopic techniques for large AO remains a matter of debate.

Adrenal oncocytoma are usually non-functional, and only about 10–20% of cases present with adrenocortical hormone production. This case presented oversecretion of androgen pathway hormones with elevated plasma levels of DHEAS, testosterone, and estradiol. As has been shown in published reports, androgen-secreting lesions increase the likelihood of adrenal malignancy, a finding considered to be suspicious by the authors (16, 17). In literature, very few cases of virilizing AO have been reported in female children or women; onset symptomatology reported was pseudoprecocious puberty, hirsutism, or deepening of the voice (18–20). Another clinical presentation of functional AO may be Cushing’s syndrome (21) or pheochromocytoma-like syndrome (22, 23). In few cases, a co-secretion of cortisol with testosterone and aldosterone was reported (24, 25).

Histopathology characteristics of this case meet the typical features of AO, assessed by the Lin–Weiss–Bisceglia criteria. If one or more of the major criteria (a mitotic rate of more than 5 mitoses per 50 high power fields, any atypical mitosis or venous invasion) is detected, the tumor is considered malignant. Large-sized tumors (>10 cm and/or >200 g), necrosis, capsular, and sinusoidal invasion are minor criteria to define an oncocytic tumor as borderline/uncertain malignant potential (1). Our patient presented a benign oncocytic tumor, in which situation, the prognosis seems excellent, and no adjuvant treatment was recommended after radical surgery. Considering the benign pattern of AO, we do not believe the outcome of PTC to be worse for this patient. Regular imaging follow-up at 12 and 18 months for a minimum of 5 years is planned as well as biochemical follow-up for this adrenal lesion of uncertain clinical behavior.

To our knowledge, no hereditary syndrome is known comprising AO and PTC. The AO was BRAF^V600E^ negative, and no somatic mutation was found, while the PTC was BRAF^V600E^ positive. However, we did not perform germline mutation analysis. In literature, two cases of functional adrenal adenoma (5, 6) and two cases of ACC presenting simultaneously with PTC have been reported (7, 8). Wanta et al. (8) performed germline mutation analysis and found wild-type *p53* and no mutation in the *Menin* gene, thus concluding the absence of a hereditary cancer syndrome. Only one case of borderline malignant non-functional AO with simultaneous PTC has been reported (26). However, no mutation analyses had been performed.

## Concluding Remarks

In conclusion, we present the first case of an androgen-secreting AO with concomitant PTC. We probably observed the simultaneous incidental discovery of two independent endocrine neoplasms. Regular and long-term imaging with biochemical follow-up is recommended, since the outcome and clinical behavior of AO remain uncertain.

## Ethics Statement

The patient provided written informed consent for research participation and for the publication of indirectly identifiable data.

## Author Contributions

Wrote the first draft of the manuscript: MPod. Contributed to the writing of the manuscript: SS. Made contributions to the acquisition of the clinical data: MPod, MPu, CT, MPro, FT, and SS. Performed pathological analyses: MPu. Agreed with manuscript results and conclusions: MPod, MPu, CT, MPro, FT, and SS. Made critical revisions and approved final version: MPu, FT, and SS. All the authors revised and approved the final manuscript and agreed to be accountable for the content of the work.

## Conflict of Interest Statement

The authors declare that the research was conducted in the absence of any commercial or financial relationships that could be construed as a potential conflict of interest. The reviewer JL and handling editor declared their shared affiliation.
