# Supplementary material for: Oncocytic Adrenocortical Neoplasm with Concomitant Papillary Thyroid Cancer
Source: Front Endocrinol (Lausanne). 2018 Jan 22;8:384. doi: 10.3389/fendo.2017.00384 (PMC5786566; doi:10.3389/fendo.2017.00384)
Supplement: Supplementary file 1 [file Table_1.DOCX]

Table S1. List of 50 target genes used for Ion AmpliSeq Cancer Hotspot Panel v2

*ABL1 EGFR GNAS KRAS PTPN11*

*AKT1 ERBB2 GNAQ MET RB1*

*ALK ERBB4 HNF1A MLH1 RET*

*APC EZH2 HRAS MPL SMAD4*

*ATM FBXW7 IDH1 NOTCH1 SMARCB1*

*BRAF FGFR1 JAK2 NPM1 SMO*

*CDH1 FGFR2 JAK3 NRAS SRC*

*CDKN2A FGFR3 IDH2 PDGFRA STK11*

*CSF1R FLT3 KDR PIK3CA TP53*

*CTNNB1 GNA11 KIT PTEN VHL*
